# Age Matching Is Essential for the Study of Cerebrospinal Fluid sTREM2 Levels and Alzheimer’s Disease Risk: A Meta-Analysis

**DOI:** 10.3389/fnagi.2021.775432

**Published:** 2021-11-12

**Authors:** Shimin Hu, Na Pan, Chunyan Liu, Yuping Wang, Tingting Zhang

**Affiliations:** ^1^Department of Neurology, Xuanwu Hospital, Capital Medical University, Beijing, China; ^2^Beijing Key Laboratory of Neuromodulation, Beijing, China; ^3^Institute of Sleep and Consciousness Disorders, Center of Epilepsy, Beijing Institute for Brain Disorders, Capital Medical University, Beijing, China

**Keywords:** Alzheimer’s disease, sTREM2, age matching, meta-analysis, cerebrospinal fluid

## Abstract

**Background:** Both the genetic and pathological studies link Alzheimer’s disease (AD) to the triggering receptor expressed on myeloid cells 2 (TREM2). A large number of studies have explored the value of cerebrospinal fluid (CSF) soluble TREM2 (sTREM2) levels as a biomarker for the diagnosis and prediction of AD; however, the findings are inconsistent. We aimed to review the studies that investigated the association of CSF sTREM2 levels and AD risk, and to provide the recommendations for future research.

**Methods and Results:** A systematic literature search was performed using the MEDLINE, EMBASE, and Web of Science (all databases) databases. The meta-analysis for the association between the CSF sTREM2 levels and AD risk included 15 studies (17 comparisons) with a total of 1,153 cases and 1,626 controls. The total results showed that the higher CSF sTREM2 levels and AD risk were associated [standardized mean difference (SMD) = 0.428, 95% *CI* (0.213, 0.643), *I*^2^ = 81.1%]. However, the analysis of the subgroup of “age difference ≤ 2 years” indicated that sTREM2 was not associated with AD [SMD = 0.090, 95% *CI* (−0.092, 0.272), *I^2^* = 27.4%] and had a significantly lower heterogeneity. Combining the results of the “age difference of 5–10 years” [SMD = 0.497, 95% *CI* (0.139, 0.855), *I*^2^ = 82.5%] and “age difference > 10 years” [SMD = 0.747, 95% *CI* (0.472, 1.023), *I*^2^ = 50.0%] subgroups showed that the difference in CSF sTREM2 between the AD and control groups was positively correlated with the age difference. A meta-regression analysis showed that the age difference can explain 33.4% of the between-study variance. By conducting further subgroup analyses of the five age-matched studies (495 cases and 364 controls) according to the measurement method, and whether inclusion criteria containing the requirement for pathological evidence of AD, no changes were observed in the corresponding pooled SMD or in the significance of the results. The meta-analysis result of “age difference ≤ 2 years” group was robust in the sensitivity analysis.

**Conclusion:** The available high-quality evidence does not yet support an association between the CSF sTREM2 levels and AD risk. Age matching between the patients with AD and cognitively unimpaired controls was a major influencing factor in the results.

## Introduction

Alzheimer’s disease (AD), as a clinically heterogeneous and complex neurodegenerative disease, is considered the most common type of dementia and induces a heavy burden to the patients and families. The onset of AD is a continuous progressive process that from an asymptomatic state to mild cognitive impairment and to clinical diagnosis of dementia (Morris, 2005). Early risk estimation and identification are essential to prevent or slow down the development of AD, which means the reliable biomarkers are necessary.

Typical pathological features in the brain of the patients with AD are beta-amyloid (Aβ) plaques, intracellular neurofibrillary tangles consisting of hyper-phosphorylated tau fibrils, degeneration of neurons, and loss of synapses, neuroinflammation, and glial activation (Bronzuoli et al., 2016). As the main immune cells in the brain, microglia have been shown to be the important modulators of AD. It is reported that the initial activation of microglia induces phagocytosis of Aβ, prevents oligomer formation, and limits the neurotoxicity of Aβ deposited in plaques; however, its continuous activation and further inflammatory response might accelerate the neurodegeneration (Nordengen et al., 2019). Currently, the inflammatory mechanism of AD has been demonstrated. However, in contrast to the use of classical fluid biomarkers of Aβ and tau proteins, the reliable inflammatory markers in the diagnosis of AD are lacking.

A triggering receptor expressed on myeloid cells 2 (TREM2) is a transmembrane protein with V-immunoglobulin extra-cellular domains and cytoplasmic tails (Bouchon et al., 2000). In the brain, a TREM2 is almost exclusively expressed by microglia (Sessa et al., 2004). A TREM2 participates in microglia functions, such as survival, proliferation, clustering around plaques, as well as phagocytosis of apoptotic cells and myelin debris (Ulland et al., 2017). Two genome-wide association studies based on different populations published in 2013 showed that a variant (p.R47H) in *TREM2* increased the risk of AD by almost threefold (Guerreiro et al., 2013; Jonsson et al., 2013). It is one of the strongest genetic risk factors for AD following the amyloid-beta precursor protein (*APP*), presenilin-1 (*PSEN1*), and apolipoprotein E (*APOE*). Soluble TREM2 (sTREM2) is generated and released into the extracellular milieu through cleavage of full-length TREM2 by disintegrin and metalloproteinase domain-containing protein 17 (ADAM17) and ADAM10 (Wu et al., 2015; Ulland and Colonna, 2018). A large number of studies have explored the value of cerebrospinal fluid (CSF) sTREM2 levels as a biomarker for the diagnosis and prediction of AD. However, previous studies have reported the conflicting results. A meta-analysis published in 2018 analyzed seven studies and concluded that the CSF sTREM2 levels in the AD cases were increased compared with controls (Liu et al., 2018). However, nine additional relevant articles were published since then, and more than half of the literature showed that the CSF sTREM2 levels are not associated with AD risk (Brosseron et al., 2018; Heywood et al., 2018; Deming et al., 2019; Morenas-Rodríguez et al., 2019; Nordengen et al., 2019; Suarez-Calvet et al., 2019; Banerjee et al., 2020; Knapskog et al., 2020; Van Hulle et al., 2021). Therefore, it is necessary to update the meta-analysis.

This study summarized the studies on the association of CSF sTREM2 and AD risk, with the attempt to find the potential influence factors to interpret the considerable differences of the results reported by these studies and provide recommendations for future research. The previous studies have shown a positive association between the CSF sTREM2 levels and age (Gispert et al., 2016, 2017; Henjum et al., 2016; Piccio et al., 2016; Suarez-Calvet et al., 2016, 2019; Brosseron et al., 2018; Deming et al., 2019; Morenas-Rodríguez et al., 2019; Knapskog et al., 2020). Meanwhile, age is the strongest predictor of AD. To reveal the value of CSF sTREM2 levels in the diagnosis and prediction of AD, the positive results from the studies with age-matched strategy are more credible. Thus, we particularly analyzed the impact of the age difference between the patients with AD and cognitively unimpaired controls on the conclusion of the association between CSF sTREM2 level and AD risk.

## Methods

### Literature Search

The databases MEDLINE, EMBASE, and Web of Science (all databases, such as Web of Science Core Collection and BIOSIS Citation Index) were searched up to April 13, 2021 to find the studies evaluating the relationship between CSF sTREM2 level and AD risk. The following keywords were used: (cerebrospinal fluid) AND (TREM2 OR sTREM2 OR “soluble trigger receptor expressed on myeloid cells 2”) AND [(Alzheimer’s disease) OR (dementia)]. All the reference lists of the resulting primary research reports and relevant reviews were manually searched to identify the additional eligible studies.

### Eligible Studies, Data Extraction, and Quality Evaluation

The eligible studies were included in this meta-analysis: (1) investigated the relationship between the CSF sTREM2 levels and AD risk; (2) included a case group of patients with AD and a group of cognitively unimpaired controls; (3) clinical criteria that used to diagnose AD were qualified; (4) provided data with the mean and SD or median and interquartile range (IQR) or median and the minimum and maximum; (5) the studies performed in humans; (6) full text in English. The articles were excluded if they measured sTREM2 concentrations in the postmortem samples, had a sample size of less than 5, or used the samples that overlapped with other studies. Information regarding the first author, year of publication, study location (country), study design, number of patients and controls, diagnostic criteria, measurement methods, the mean and SD of CSF sTREM2 levels, and information for the potential confounding factors (i.e., age) were extracted.

The quality of case-control studies and cohort studies included was evaluated based on the Newcastle-Ottawa Scale (NOS) recommended by the Agency for Healthcare Research and Quality of the United States. Comparability of the cases and controls on the basis of the design or analysis was evaluated based on whether the age and gender matched. The methodological quality of the cross-sectional studies included was assessed using an 11-item checklist which was recommended by Agency for Healthcare Research and Quality (AHRQ). The studies with NOS scores lower than 5, or AHRQ scores lower than 4 were recognized to be of inferior quality and therefore excluded.

The disputes were resolved by discussion with a third author during data extraction and quality evaluation. If the case or control groups were further divided into subgroups, the data from the subgroups were merged as *n* = *n*_1_ + *n*_2_, $\bar{x}=\frac{n_{1}\bar{x_{1}}+n_{2}\bar{x_{2}}}{n_{1}+n_{2}}$ and $\mathrm{SD}=\sqrt{\frac{\left(n_{1}-1\right)SD_{1}^{2}+\left(n_{2}-1\right)SD_{2}^{2}+\frac{n_{1}n_{2}}{n_{1}+n_{2}}\left({\bar{x}}_{1}^{2}+{\bar{x}}_{2}^{2}-2{\bar{x}}_{1}{\bar{x}}_{2}\right)}{n_{1}+n_{2}-1}}$. When a study provided medians and IQRs (instead of means and SDs), without the minimum or maximum values, we treated the medians as the means and calculated the SDs as SD = IQR/1.35 (Higgins et al., 2021). If the study provided the minimum and maximum, we imputed the means and SDs as described by Hozo et al. (2005).

### Statistical Analysis

The association between the CSF sTREM2 level and AD risk was estimated by calculating the pooled standardized mean difference (SMD) and the 95% *CI*. To analyze the potential influences of age difference at the time of lumbar puncture, measurement method, and inclusion criteria containing the requirement for pathological evidence of AD, we performed the subgroup analysis. Based on the age difference between the AD group and the control group at lumbar puncture, the subjects were divided into three subgroups: (1) age difference ≤ 2 years, (2) age difference of 5–10 years, and (3) age difference > 10 years. Based on the measurement method, the subjects were divided into three subgroups: (1) enzyme-linked immunosorbent assay (ELISA), (2) mesoscale discovery electrochemiluminescence platform-based assay (MSD) or electrochemiluminescence (ECL) immunoassay, and (3) ultra-performance liquid chromatography-tandem mass spectrometer (UPLC-MS). Additionally, based on the diagnostic criteria, the subjects were divided into two subgroups: (1) with neuropathologic verification, and (2) no relative information. The significance of the pooled SMD was determined using a *Z* test, and the level was set at *p* < 0.05.

Heterogeneity across the studies was assessed using the *Q* test and the *I*^2^ statistic. When significant heterogeneity was observed (*p* < 0.1 in the *Q* test, and *I*^2^ > 50%), a random effects model was used for pooling the data from the primary studies. A meta-regression with restricted maximum likelihood estimation (REML) was performed to assess the potentially important covariate exerting substantial impact on the between-study heterogeneity. The age difference (≤2 years, 5–10 years, and >10 years) was included in the meta-regression analysis. The sensitivity analysis was performed by sequentially excluding the individual studies to assess the stability of the results. A funnel-plot asymmetry was assessed using Egger’s linear regression test, with *p* < 0.05 representing significant publication bias. All the analyses were performed using STATA 12.0 software (Stata Corporation, College Station, TX, United States), and all the *p* values were two-tailed.

## Results

### Study Selection

With respect to the association between the CSF sTREM2 levels and AD risk, an initial search identified 184 records of the potentially relevant studies from the databases. Of these, 152 records were excluded based on their title and/or abstract: these were repetitive publications (*n* = 90), conference abstracts (*n* = 14), the reports of animal studies (*n* = 7), the reports of studies that investigated either the outcomes irrelevant to this meta-analysis (*n* = 18), the markers other than sTREM2 (*n* = 1), review or meta-analysis (*n* = 12), protocol (*n* = 1), the studies of which sTREM2 levels was not measured in CSF (*n* = 2), the reports of studies that only had *TREM2* gene information (*n* = 6), or the studies of which did not have a normal cognitive function group (*n* = 1).

**FIGURE 1:**
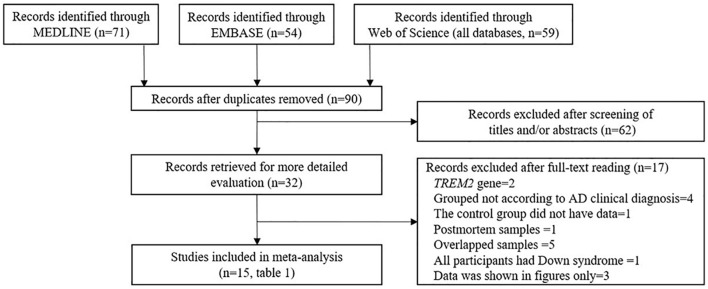
Preferred reporting items for systematic reviews and meta-analyses (PRISMA) flow diagram of the study selection process.

A further 17 articles were excluded after reviewing the full text. These were (1) *TREM2* gene study (*n* = 2), (2) the studies in which the interviewees were not grouped according to the AD clinical diagnosis criteria (*n* = 4), (3) study in which the sTREM2 levels were not detected in the control group (*n* = 1), (4) postmortem samples (*n* = 1), (5) the studies used the samples that overlapped with other studies (*n* = 5), (6) the studies in which all the participants had Down syndrome (*n* = 1), and (7) the studies in which data were shown in figures only (*n* = 3). The 15 studies (17 comparisons) that were ultimately selected for our meta-analysis included 1,153 cases and 1,626 controls (Table 1 and Supplementary Tables 1, 2); the process of study selection is shown in Figure 1.

**TABLE 1:** The detailed characteristics of all the eligible studies for the association with the cerebrospinal fluid soluble triggering receptor expressed on myeloid cells 2 (CSF sTREM2) levels and Alzheimer’s disease (AD).

Author, year	Location	Study Design	Number AD/C	AD diagnosis	Measurement method	Age AD/C	AD pathology	sTREM2 levels, ng/ml Mean (SD)		
								AD	Control	*p*
Van Hulle et al., 2021	United States	CS*	50/606	NIA-AA	ECL immunoassay	72.8/62.4	NI	9.87 (3.46)	8.01 (2.46)	< 0.001
Knapskog et al., 2020	Norway	CC	237/113	NIA-AA	ELISA	70.1/72.3	NI	9.5 (4.8)	8.8 (3.6)	NS
Banerjee et al., 2020	United Kingdom	CC	20/10	NIA-AA	MSD	62.5/62.2	Verification	6.58 (1.84)	7.96 (2.71)	NS
Deming et al., 2019	ADNI	CC	172/169	NINCDS–ADRDA	MSD	74.4/74.5	NI	4.019 (1.946)	3.988 (1.923)	NS
Nordengen et al., 2019	Norway	CC	27/36	NIA-AA	ELISA	67.5/61.1	Verification	4.8 (1.7)	3.1 (0.9)	< 0.001
Morenas-Rodríguez et al., 2019	Spain	CC	36/40	NIA-AA	ELISA	74.6/67.4	Verification	4.3 (2.2)	4.2 (2.3)	NS
Heywood et al., 2018	Italy	CC	13/15	NINCDS-ADRDA	UPLC-MS	72.1/61.0	Verification	261.7 (82.0) mol/ml	154.7 (53.1) mol/ml	< 0.001
Brosseron et al., 2018	Germany	CC	116/85	NIA-AA	MSD	74/67	Verification	4.315 (2.235)	2.994 (2.269)	0.002
Gispert et al., 2017	Spain	CC	15/49	NIA-AA	MSD	67.14/61.92	Verification	0.5866 (0.3589) relative to an internal standard	0.4235 (0.2176) relative to an internal standard	0.035
Suarez-Calvet et al., 2016	Sweden Germany Belgium Spain	CC	200/150	NIA-AA	MSD	73.8/62.4	Verification	5.33 (3.7)	3.07 (1.4)	< 0.001
Piccio et al., 2016	Italy United States	CC	73/107	NINCDS-ADRDA	ELISA	76.6/70.2	NI	1.028 (0.388)	0.832 (0.339)	0.015
Heslegrave et al., 2016	United Kingdom	CC	37/22	IWG2	UPLC-MS	70.5/69.2	Verification	0.2312 (0.0984)	0.1956 (0.0813)	0.0457
	Sweden		24/16			64.3/55.6		0.2305 (0.0970)	0.1713 (0.0652)	0.0312
Henjum et al., 2016	Sweden	CC	25/25	NINCDS-ADRDA	ELISA	76.25/61.75	NI	3.8 (2.22)	3.2 (1.63)	0.76
	Norway		29/50	NIA-AA		68/66	Verification	4.8 (2.67)	4.4 (2)	0.17
Gispert et al., 2016	Spain	CC	23/45	NIA-AA	MSD	66.78/60.98	Verification	3.34 (1.35)	2.40 (1.14)	0.004
Kleinberger et al., 2014	Germany	CC	56/88	NINCDS-ADRDA	ELISA	70.4/60.7	Verification	0.309 (0.191) relative to an internal standard	0.381 (0.174) relative to an internal standard	0.001

CC, Case-control study; CS, Cross-sectional study; NIA-AA, National Institute on Aging-Alzheimer’s Association; NINCDS–ADRDA, National Institute of Neurological and Communicative Disorders and Stroke and the Alzheimer’s Disease and Related Disorders Association; IWG2, the revised proposed International Working Group; ECL immunoassay, electrochemiluminescence immunoassay; ELISA, enzyme-linked immunosorbent assay; MSD, mesoscale discovery electrochemiluminescence platform-based assay; UPLC-MS, ultra-performance liquid chromatography-tandem mass spectrometer; NI, no information; NS, non-significant. *Although the subjects in this article were part of a cohort, the authors requested that lumbar punctures were performed within 1 year of cognitive testing. Therefore, we believed this article should more appropriately be classified as a cross-sectional study.

### Association Between the Cerebrospinal Fluid sTREM2 Levels and Alzheimer’s Disease Risk in All Eligible Comparisons

**FIGURE 2:**
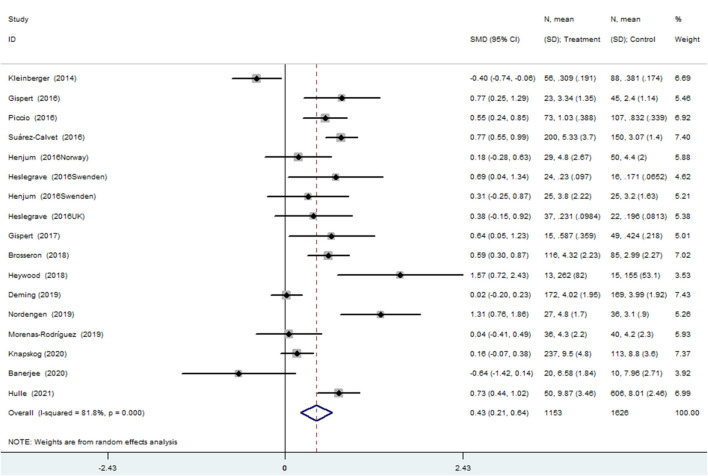
Meta-analysis for the association of the cerebrospinal fluid soluble triggering receptor expressed on myeloid cells 2 (CSF sTREM2) level with Alzheimer’s disease (AD) risk using a random effects model.

The meta-analysis for the association between the CSF sTREM2 levels and AD risk included 15 studies (17 comparisons) with a total of 1,153 cases and 1,626 controls. The total results showed that a higher CSF sTREM2 level was associated with increased AD risk [SMD = 0.428, 95% *CI* (0.213, 0.643), *I^2^* = 81.1%] (Figure 2).

**FIGURE 3:**
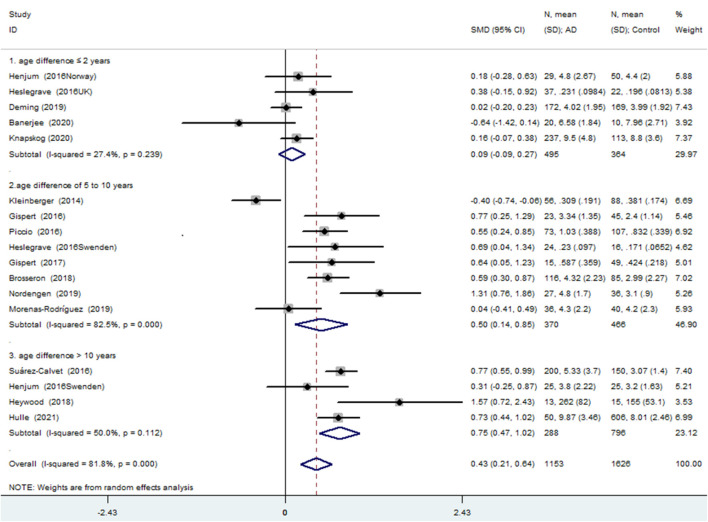
Age difference at a lumbar puncture subgroup analysis using a random effects model.

Of the 17 comparisons, four comparisons had an age difference greater than 10 years, eight comparisons had an age difference from 5–10 years, and five studies had an age difference of 2 years or less. When distinguishing the subgroups by age difference, the heterogeneity decreased substantially in both the subgroup of “age differences ≤ 2 years” (*I*^2^ = 27.4%) and the subgroup of “age differences > 10 years” (*I*^2^ = 50.0%). An analysis of the subgroup of “age difference ≤ 2 years” indicated that sTREM2 was not associated with AD [SMD = 0.090, 95% *CI* (−0.092, 0.272), *I^2^* = 27.4%]. An analysis of the subgroup of “age difference of 5–10 years” [SMD = 0.497, 95% *CI* (0.139, 0.855), *I*^2^ = 82.5%], and the subgroup of “age difference > 10 years” [SMD = 0.747, 95% *CI* (0.472, 1.023), *I*^2^ = 50.0%] showed that the CSF sTREM2 levels were associated with AD. The results indicated that the greater the difference in age, the greater the difference in CSF sTREM2 levels between the case group and the control group (Figure 3 and Table 2). The meta-regression analysis showed that age difference could explain 33.4% of the between-study variance (coefficient = 0.354, SE = 0.144, *t* = 2.45, and *p* = 0.027).

**TABLE 2:** Summary of different comparative results of the CSF sTREM2 level with AD risk in all the eligible comparisons.

Category		No. of comparisons	No. of cases	No. of controls	SMD (95% CI)	Z	*p* Value	I^2^%	*p*_*het*_
Overall		17	1,153	1,626	0.428 (0.213, 0.643)	3.90	< 0.001	81.8	< 0.001
Age difference	≤2 years	5	495	364	0.090 (−0.092, 0.272)	0.97	0.332	27.4	0.239
	5–10 years	8	370	466	0.497 (0.139, 0.855)	2.72	0.007	82.5	< 0.001
	>10 years	4	288	796	0.747 (0.472, 1.023)	5.31	< 0.001	50.0	0.112
Measurement method	ELISA	7	483	459	0.280 (−0.057, 0.616)	1.63	0.103	81.9	< 0.001
	MSD or ECL immunoassay	7	596	1,114	0.463 (0.144, 0.781)	2.85	0.004	84.1	< 0.001
	UPLC-MS	3	74	53	0.810 (0.179, 1.441)	2.52	0.012	62.5	0.069
AD pathology	With neuropathologic verification	15*	849	1,316	0.477 (0.236, 0.718)	3.87	< 0.001	81.0	< 0.001
	No relative information	5	557	1,020	0.342 (0.060, 0.624)	2.38	0.017	79.2	0.001

SMD, standardized mean difference; p_het_, p value for heterogeneity. *Compared with the 17 comparisons used for the overall analysis, three additional comparisons were added for the subgroup analysis of AD pathology: specific information was described in the results section.

Subgroup analysis of the 17 comparisons was carried out according to the measurement method and the heterogeneity within each subgroup remained high. The results suggested that the measurement methods might impact the results of the difference in the CSF sTREM2 levels between the patients with AD and controls [ELISA: SMD = 0.280, 95% *CI* (−0.057, 0.616), *I*^2^ = 81.9%; MSD or ECL immunoassay: SMD = 0.463, 95% *CI* (0.144, 0.781), *I*^2^ = 84.1%; UPLC-MS: SMD = 0.810, 95% *CI* (0.179, 1.441), *I^2^* = 62.5%; Supplementary Figure 1 and Table 2].

Subgroup analysis of the 20 comparisons according to whether the study diagnostic criteria contained AD pathology was conducted. In addition, the data showed a high degree of heterogeneity within each subgroup. The 20 comparisons included the 17 comparisons used for the overall analysis and three additional comparisons. Among the three additional comparisons, two studies (Knapskog et al., 2020; Van Hulle et al., 2021) had group comparisons after further confirmation of AD pathology in addition to the group comparisons according to the clinical AD diagnosis; another one study (Suarez-Calvet et al., 2019) used the same ADNI database as the study by Deming et al. (2019) but had smaller sample size and confirmed AD pathology (as shown in Supplementary Table 3). The results suggested that the more demanding the diagnostic criteria, the greater the difference between the two CSF sTREM2 groups [with neuropathologic verification: SMD = 0.477, 95% *CI* (0.236, 0.718), *I*^2^ = 81.0%; no relative information: SMD = 0.342, 95% *CI* (0.060, 0.624), *I*^2^ = 79.2%; Supplementary Figure 2 and Table 2].

### Association Between the Cerebrospinal Fluid sTREM2 Levels and Alzheimer’s Disease Risk in “Age Difference ≤ 2 Years” Group

A total of five studies with approximately equal age between the patients with AD and cognitively unimpaired controls were included. All of these were the case-control studies involving 495 patients with AD and 364 cognitively unimpaired controls. Two studies were published in 2016 and the other three were published after 2019. Four studies were carried out in Europe and one was in the United States. The mean age of participants in the four studies ranged from 68 to 74 years and the mean age in the remaining study was 62.5 years. In terms of method, two studies used ELISA, two used MSD, and one used UPLC-MS for the measurement of the sTREM2 levels. Three articles had restrictions on the pathological status of AD in both the groups and two had no restrictions.

The meta-analysis of the studies with an age difference within 2 years showed that the CSF sTREM2 levels were not associated with AD and there was little heterogeneity between the studies [SMD = 0.090, 95% *CI* (−0.092, 0.272), *I^2^* = 27.4%]. Neither the subgroup analysis of the five articles distinguished by the measurement method [ELISA: SMD = 0.161, 95% *CI* (−0.040, 0.363), *I*^2^ = 0%; others: SMD = 0.001, 95% *CI* (−0.413, 0.415), *I^2^* = 56.2%; Supplementary Figure 3 and Table 3] nor by whether the study diagnostic criteria contained AD pathology [with neuropathologic verification: SMD = 0.103, 95% *CI* (−0.188, 0.394), *I^2^* = 35.9%; without neuropathologic verification: SMD = 0.083, 95% *CI* (−0.071, 0.237), *I^2^* = 0%; Supplementary Figure 4 and Table 3] showed association between the CSF sTREM2 level and AD risk.

**TABLE 3:** Summary of different comparative results of CSF sTREM2 level with AD risk in “age difference ≤ 2 years” group.

Category		No. of comparisons	No. of cases	No. of controls	SMD (95% CI)	Z	*p* Value	I^2^%	*p*_*het*_
Age difference ≤ 2 years		5	495	364	0.090 (−0.092, 0.272)	0.97	0.332	27.4	0.239
Measurement method	ELISA	2	266	163	0.161 (−0.040, 0.363)	1.57	0.117	0	0.941
	Others	3	229	201	0.001 (−0.413, 0.415)	< 0.01	0.998	56.2	0.102
AD pathology	With neuropathologic verification	4*	166	204	0.103 (−0.188, 0.394)	0.70	0.487	35.9	0.197
	No relative information	2	409	282	0.083 (−0.071, 0.237)	1.05	0.293	0	0.370

SMD, standardized mean difference; p_het_, p value for heterogeneity. *Compared with the five comparisons used for the “age difference ≤ 2 years” analysis, one additional comparison was added for the subgroup analysis of AD pathology: specific information was described in the results section.

### Publication Bias and Sensitivity Analysis

**FIGURE 4:**
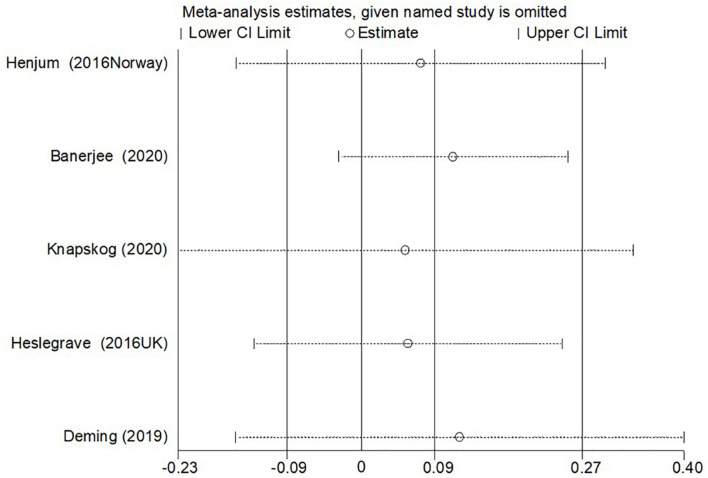
The results of the sensitivity analysis of the CSF sTREM2 level with AD risk in the studies with approximately equal age between the AD group and control group.

**FIGURE 5:**
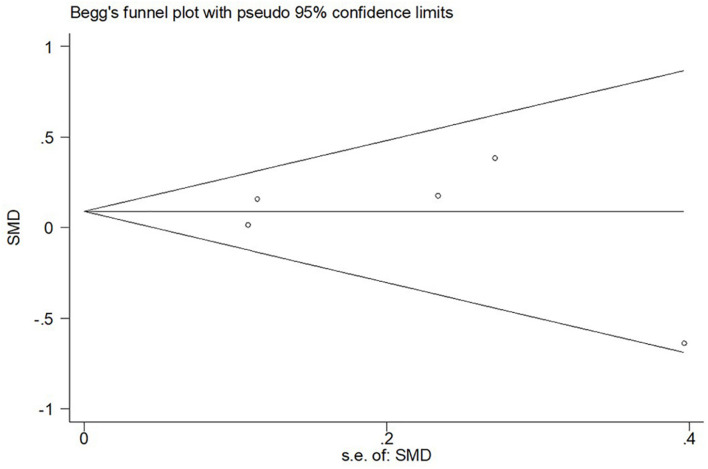
Begg’s funnel plot for publication bias test of CSF sTREM2 level with AD risk in the studies with approximately equal age between the AD group and control group.

In the sensitivity analysis of the meta-analysis of “age difference ≤ 2 years” group, one eligible study was excluded at a time to assess the influence of each dataset on the pooled SMD. We observed no changes in the corresponding pooled SMD or in the significance of the results (Figure 4), which indicated that our results were significantly stable to the study-selection process. The publication bias was assessed using the Begg’s funnel plot and Egger’s test (Figure 5). The results of Begg’s funnel plot and the modified Egger linear regression test showed no publication bias (*Z* = −0.24, *p* = 1.000; *t* = −0.34, *p* = 0.753).

In addition, the meta-analysis of 17 comparisons showed robust results with no publication bias (Begg’s test: *Z* = 0.45, *p* = 0.650; Egger’s test: *t* = 0.62, *p* = 0.542; Supplementary Figures 5, 6).

## Discussion

### Main Findings

Our meta-analysis shows that the higher CSF sTREM2 level is related to AD risk, but this association cannot be shown in the studies in which age was matched or showed a similar distribution in the AD and control groups. Greater age differences between the case andcontrol groups would result in a larger differencein CSF sTREM2 levels between groups.

### Interpretation

The association between TREM2 and cognitive impairment was first identified through the studies examining the mutations in the patients with Nasu-Hakola disease (NHD) (Paloneva et al., 2002). The patients with NHD are asymptomatic through early adulthood, present with neuropsychiatric symptoms in their fourth decades which are characterized by frontal lobe syndrome and progressive dementia (Montalbetti et al., 2004). The number of microglia was lower in various regions of the brain in the TREM2-deficient mice; and this finding was only confirmed in the aging mice (1–2 years old) (Poliani et al., 2015). Complete lack of *TREM2* affects microglia function in the brain. However, additional risk factors, such as increased age, are probably necessary to elicit neuropsychiatric pathology, at least in mice (Ulland and Colonna, 2018). In neuropathologically normal human brain, the expression of TREM2 is found to be elevated with aging (Forabosco et al., 2013).

Both the genetic and pathological studies link AD to TREM2. Increased AD risk is thought to be mediated through the reduced TREM2 function (Qin et al., 2021). The TREM2 surface expression declines upon activation of the myeloid cells with the toll-like receptor ligands or inflammatory cytokines. This phenomenon is caused at least in part by the sequential cleavage of membrane-bound TREM2 by proteases that release sTREM2 (Ulland and Colonna, 2018). The sTREM2 levels in CSF could depend on the synthesis rate of TREM2 in largely microglia and peripherally derived macrophages, transport to the cell surface of TREM2, shedding and degradation of the sTREM2 fragment (Henjum et al., 2016). The positive correlation between the CSF sTREM2 level and age is validated in the patients with clinical AD (both MCI and dementia) and clinical cognitively unimpaired individuals, and in the patients with neuropathologically confirmed AD and neuropathologically confirmed cognitively unimpaired individuals (Gispert et al., 2016, 2017; Henjum et al., 2016; Piccio et al., 2016; Suarez-Calvet et al., 2016, 2019; Brosseron et al., 2018; Deming et al., 2019; Morenas-Rodríguez et al., 2019; Knapskog et al., 2020). One Norwegian study with relatively small sample size (27 patients with AD and 36 controls) concluded that the CSF sTREM2 levels were independent of age (Nordengen et al., 2019). In our meta-analysis, the overall analysis showed positive association between the CSF sTREM2 level and AD risk; however, when we only included studies with similar age (age difference ≤ 2 years) between the patients with AD and controls, no association was found between the CSF sTREM2 and AD risk. The result of “age difference ≤ 2 years” subgroup meta-analysis was robust in the sensitivity analysis. Moreover, the heterogeneity between the studies in “age difference ≤ 2 years” subgroup was significantly lower than that in all studies included. These suggest that the negative results of “age difference ≤ 2 years” subgroup should not be ignored. In addition, combining the results of the “age difference of 5–10 years” and “age difference > 10 years” subgroups showed that the difference in CSF sTREM2 between the AD and control groups was positively correlated with the age difference. The large age difference between the AD and control groups is more likely to account for the different CSF sTREM2 levels in the two groups than the association of sTREM2 with AD risk. In addition to the issue of age matching, the mean age of the control group in most of the available studies was less than 65 years old, especially in the studies with a large age difference between the two groups. This makes the results of the overall analysis potentially subject to selection bias. Overall, for future studies we strongly recommend that the age matching between the two groups should be used as an inclusion criterion and that the age of the control group should be fully considered.

An analysis to investigate whether the diagnostic criteria affected the result revealed that the greater difference of CSF sTREM2 between the AD and control groups was shown in the subgroup of studies contained pathological diagnosis. The 95% *CI* of pooled SMD of the subgroup in which the diagnostic criteria were only clinical symptoms was 0.06–0.62, which was very close to “no association.” This suggests that the level of CSF sTREM2 may have a higher value as a biomarker for AD when based on pathological diagnosis. However, it must be noted that the studies used uniform pathological diagnosis index and the data showed a high degree of heterogeneity. The reliability of this conclusion needs further study. On the other hand, the clinical diagnosis focusing on the syndromes is still the routine diagnostic strategy because of good accuracy and the limited standardization and utility of biomarkers (Knopman et al., 2001). The value of the CSF sTREM2 level as a complementary diagnostic marker in the clinical diagnosis may be limited and requires further investigation.

In this study, when the effect of the measurement methods on the results was analyzed, different results were found for different measurement methods. Negative result was shown in the group of studies using ELISA, the simplest and most practical measurement method. Practicality and standardization of CSF sTREM2 as a complementary marker for the clinical diagnosis are yet to be investigated.

This study has certain limitations. First, the heterogeneity within the studies included in the overall analysis was high; therefore, the overall result needs to be interpreted with caution. Second, a total of five studies with approximately equal age between the AD group and the control group were included, involving 495 patients in the AD group and 364 in the control group. It will be necessary to update this meta-analysis in the future when more high-quality literature appears. Last, our study only included the articles written in English, which might have resulted in a language bias.

## Conclusion

The available high-quality evidence does not support an association between the CSF sTREM2 levels and AD risk. The matching of age between the patients with AD and cognitively unimpaired controls was a major influencing factor in the results.

## Data Availability Statement

The original contributions presented in the study are included in the article/Supplementary Material, further inquiries can be directed to the corresponding author/s.

## Author Contributions

SH and NP have searched the databases and extracted the information. YW reviewed the processes of literature search and data extraction. SH did the statistical analysis. SH and CL drafted the manuscript. TZ has revised and expanded the manuscript. All the authors read and approved the final manuscript.

## Conflict of Interest

The authors declare that the research was conducted in the absence of any commercial or financial relationships that could be construed as a potential conflict of interest.

## Publisher’s Note

All claims expressed in this article are solely those of the authors and do not necessarily represent those of their affiliated organizations, or those of the publisher, the editors and the reviewers. Any product that may be evaluated in this article, or claim that may be made by its manufacturer, is not guaranteed or endorsed by the publisher.
