# Supplementary material for: Age Matching Is Essential for the Study of Cerebrospinal Fluid sTREM2 Levels and Alzheimer’s Disease Risk: A Meta-Analysis
Source: Front Aging Neurosci. 2021 Nov 12;13:775432. doi: 10.3389/fnagi.2021.775432 (PMC8632715; doi:10.3389/fnagi.2021.775432)
Supplement: Supplementary file 1 [file Data_Sheet_1.pdf]

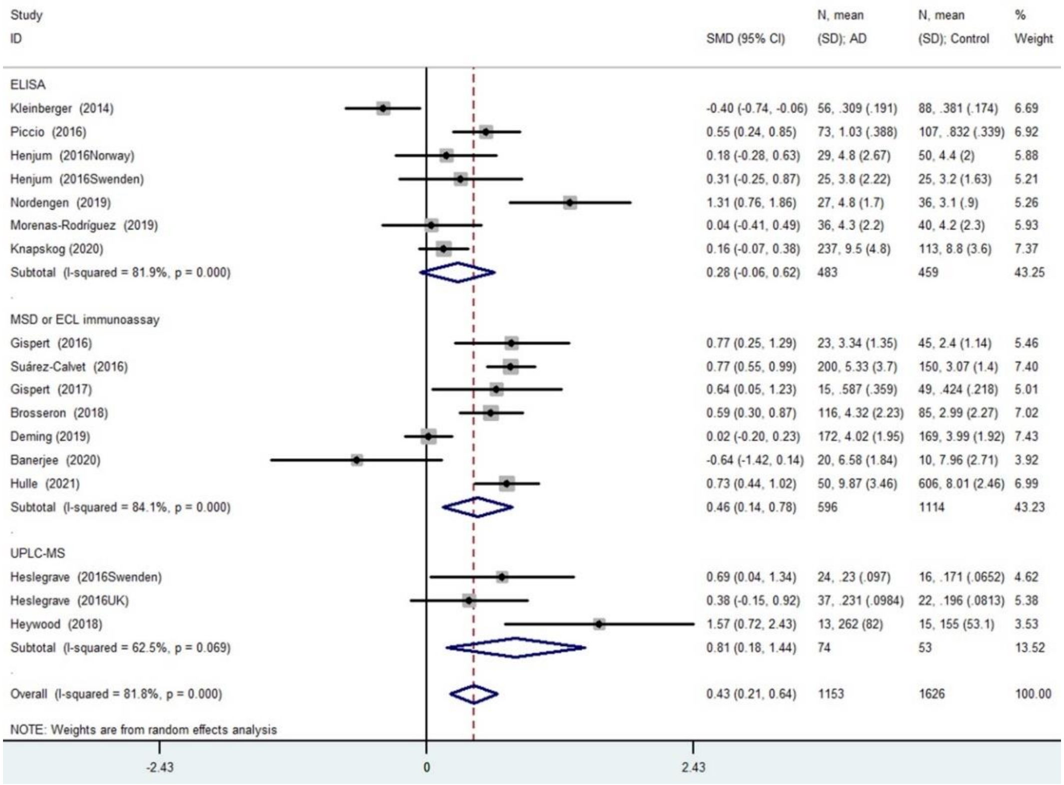

Supplementary Figure 1. Measurement method subgroup analysis using a random effects model.

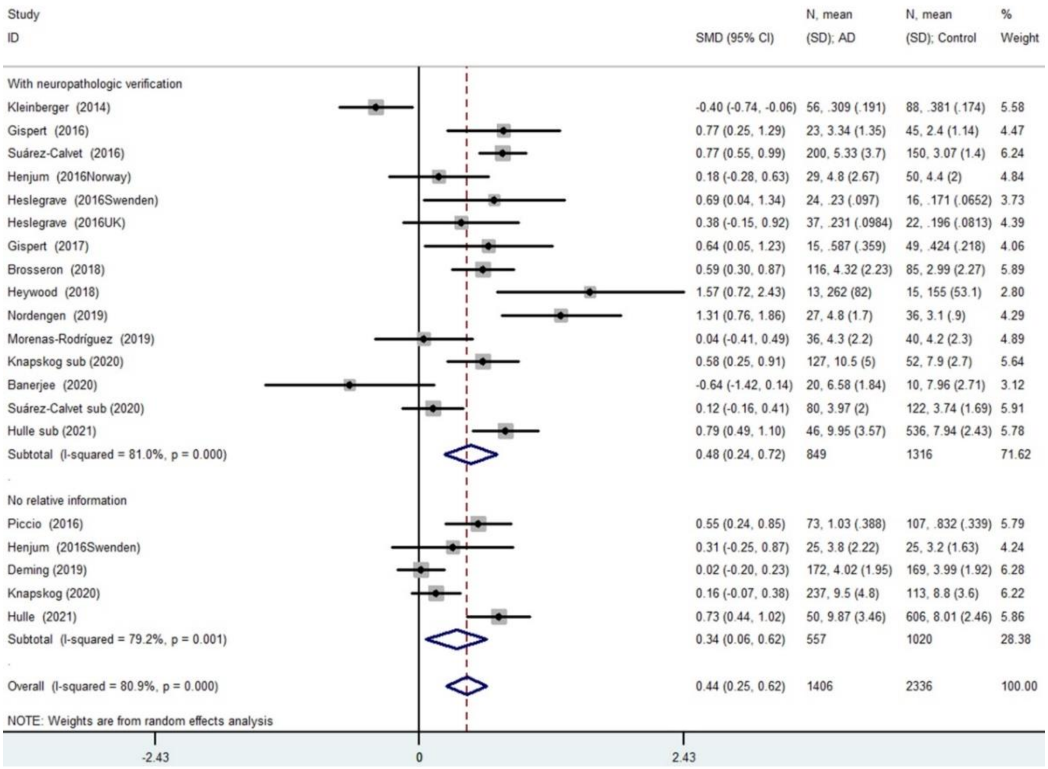

Supplementary Figure 2. Inclusion criteria containing the requirement for pathological evidence of AD subgroup analysis using a random effects model.

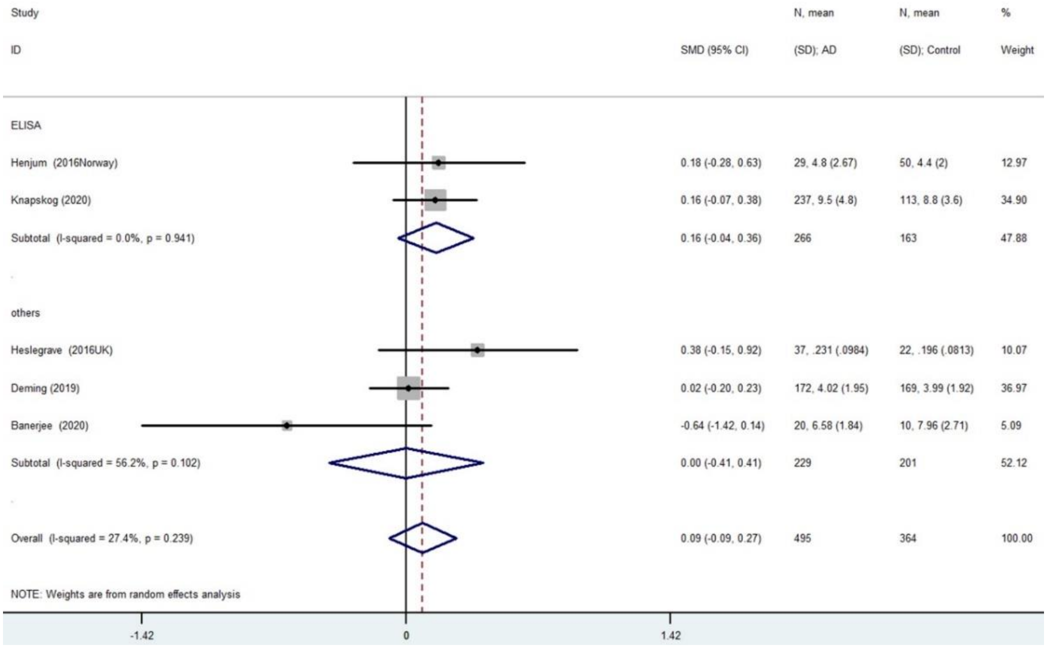

Supplementary Figure 3. Measurement method subgroup analysis using a random effects model in studies with approximately equal age between AD group and control group.

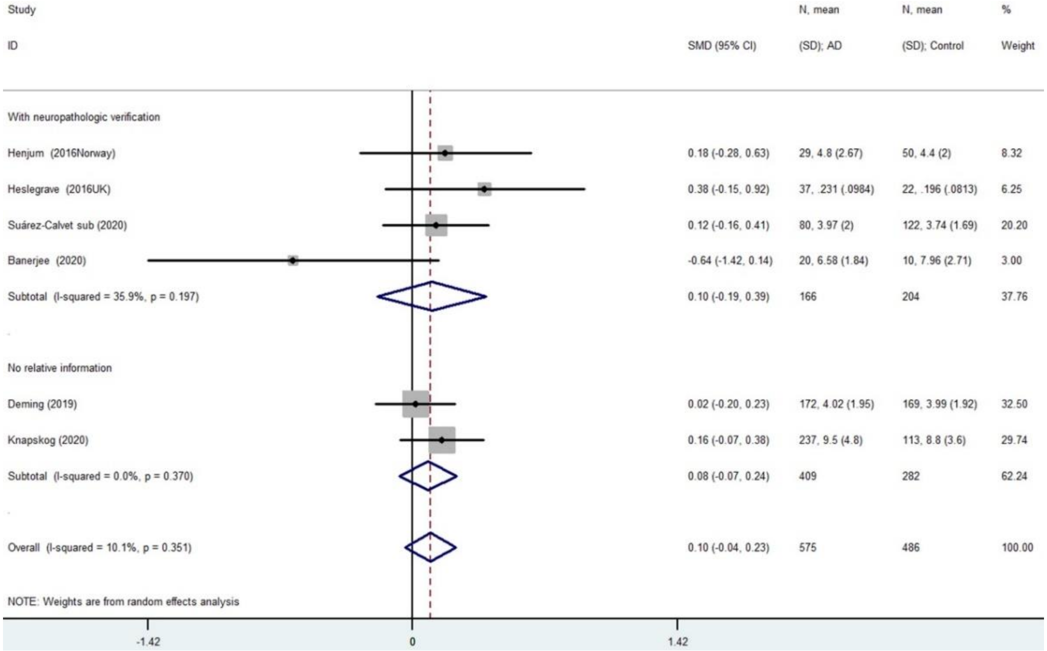

Supplementary Figure 4. Inclusion criteria containing the requirement for pathological evidence of AD subgroup analysis using a random effects model in studies with approximately equal age between AD group and control group.

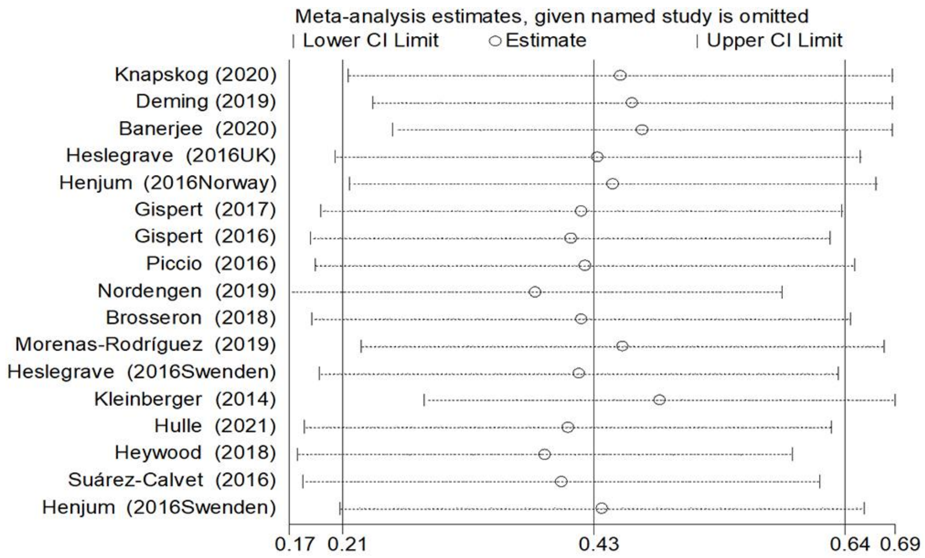

Supplementary Figure 5. The results of sensitivity analysis of CSF sTREM2 level with AD risk.

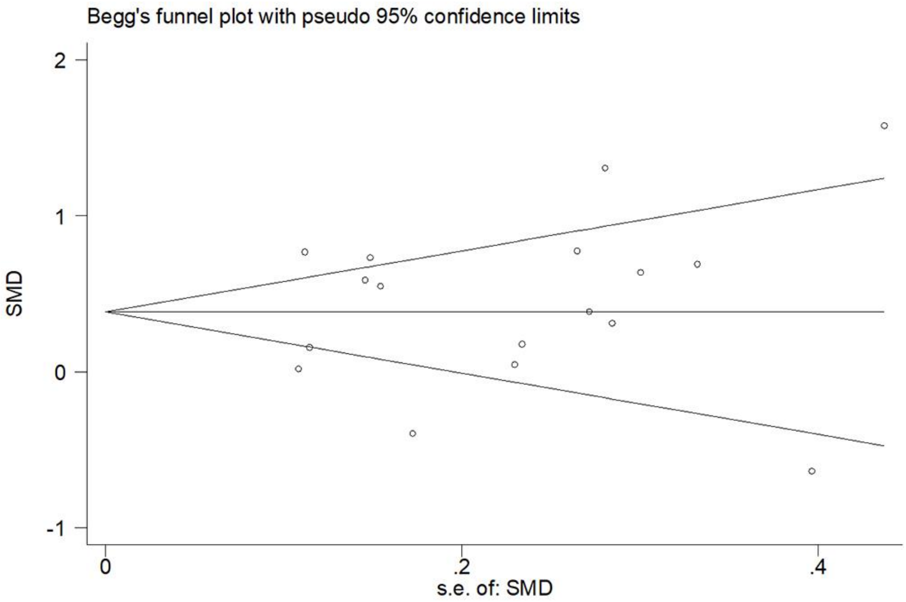

Supplementary Figure 6. Begg’s funnel plot for publication bias test of CSF sTREM2 level with AD risk.

Supplementary Table 1 The quality assessment of the included studies based on scores of Newcastle-Ottawa Scale (case-control study version)

Author, year	Case definition adequate	Representative-ness of the cases	Selection of controls (selected from different diseases or multicenter)	Definition of controls	Comparability of cases and controls (age and gender)	Ascertainment of exposure	Same method of ascertainment for cases and controls	Non-Response rate	Total quality scores
Knapskog et al., 2020	1	1	1	1	1	1	1	NI	7
Banerjee et al., 2020	1	0	1	1	2	1	1	NI	7
Deming et al., 2019	1	1	1	1	2	1	1	NI	8
Nordengen et al., 2019	1	0	1	1	1	1	1	NI	6
Morenas-Rodriguez et al., 2019	1	0	1	1	1	1	1	NI	6
Heywood et al., 2018	1	0	0	1	1	1	1	NI	5
Brosseron et al., 2018	1	0	1	1	0	1	1	NI	5
Gispert et al., 2017	1	0	0	1	1	1	1	NI	5

Suarez-Calvet et al., 2016	1	1	1	1	1	1	1	NI	7
Piccio et al., 2016	1	1	1	1	1	1	1	NI	7
Heslegrave et al., 2016 (UK)	1	0	0	1	2	1	1	NI	6
Heslegrave et al., 2016 (Sweden)	1	0	0	1	1	1	1	NI	5
Henjum et al., 2016 (Sweden)	1	0	0	1	1	1	1	NI	5
Henjum et al., 2016 (Norway)	1	0	1	1	1	1	1	NI	7
Gispert et al., 2016	1	0	0	1	1	1	1	NI	5
Kleinberger et al., 2014	1	1	0	1	1	1	1	NI	6

Supplementary Table 2 The quality assessment of the included studies based on scores of 11-item checklists which was recommended by Agency for Healthcare Research and Quality (AHRQ)

Author, year	Define the source of information	List inclusion and exclusion criteria for exposed and unexposed subjects (cases and controls) or refer to previous publications	Indicate time period used for identifying patients	Indicate whether or not subjects were consecutive if not population-based	Indicate if evaluators of subjective components of study were masked to other aspects of the status of the participants	Describe any assessments undertaken for quality assurance purposes	Explain any patient exclusions from analysis	Describe how confounding was assessed and/or controlled	Explain how missing data were handled in the analysis	Summarize patient response rates and completeness of data collection	Clarify what follow-up, if any, was expected and the percentage of patients for which incomplete data or follow-up was obtained
Van Hulle et al., 2021	1	1	NI	NI	1	1	1	1	1	NI	NI
Suarez-Calvet et al., 2019	1	1	NI	NI	1	1	1	1	1	NI	NI

Supplementary Table 3 Additional Literature Information for AD Pathology Subgroups.

Author, year	Location	Study Design	Number AD/C	AD diagnosis	Measurement method	Age AD/C	AD pathology	sTREM2 levels, ng/ml Mean(SD)			Data overlapped with the following study
								AD	Control	*p*	
Van Hulle et al., 2021	USA	CS*	46/536	NIA-AA	ECL immunoassay	72.3/61.6	Confirmed	9.95(3.57)	7.94(2.43)	<0.001	Hulle et al., 2021
Knapskog et al., 2020	Norway	CC	127/52	NIA-AA	ELISA	NI	Confirmed	10.5(5.0)	7.9(2.7)	<0.001	Knapskog et al., 2020
Suarez-Calvet et al., 2019	ADNI	CS	80/122	NIA-AA	MSD	74.1/72.5	Confirmed	3.967(2.000)	3.741(1.690)	NS	Deming et al., 2019

CC: Case-control study; CS: Cross-sectional study; NIA-AA: National Institute on Aging-Alzheimer's Association; ECL immunoassay: Electrochemiluminescence immunoassay; ELISA: Enzyme-linked immunosorbent assay; MSD: Mesoscale Discovery electrochemiluminescence platform-based assay; NI: No information; NS: Nonsignificant.

* Although the subjects in this article were part of a cohort, the authors requested that lumbar punctures were performed within 1 year of cognitive testing. Therefore, we believed this article should more appropriately be classified as a cross-sectional study.
